# Second Salvage Autologous Hematopoietic Stem Cell Transplantation in Patients with Relapsed/Refractory Multiple Myeloma in the Era of Novel Agents: Results of the KMM2301 Study

**DOI:** 10.3390/cancers18030471

**Published:** 2026-01-30

**Authors:** Jongheon Jung, Ji Hyun Lee, Sung-Hyun Kim, Jae Hoon Lee, Kwai Han Yoo, Young Rok Do, Ho-jin Shin, Kihyun Kim, Sang Eun Yoon, Dok Hyun Yoon, Hyungwoo Cho, Hye Jin Kang, Ja Min Byun, Jae-Cheol Jo, Seung-Shin Lee, Won Sik Lee, Je-Jung Lee, Sung-Hoon Jung, Myung-Won Lee, Jun Ho Yi, Ju-Hyun Park, Chang-Ki Min, Hyeon-Seok Eom

**Affiliations:** 1 Center for Hematologic Malignancy, National Cancer Center, 323 Ilsan-ro, Ilsandong-gu, Goyang 10408, Republic of Korea; jhjung@ncc.re.kr; 2 Division of Hematology-Oncology, Department of Internal Medicine, Dong-A University College of Medicine, Busan 49201, Republic of Korea; hidrleejh12@gmail.com (J.H.L.); kshmoon@dau.ac.kr (S.-H.K.); 3 Division of Hematology, Gachon University College of Medicine Gil Medical Center, Incheon 21565, Republic of Korea; jhlee@gilhospital.com (J.H.L.); khyoo@gilhospital.com (K.H.Y.); 4 Department of Hemato-Oncology, Keimyung University, Dongsan Medical Center, Daegu 42601, Republic of Korea; dyr1160@dsmc.or.kr; 5 Division of Hematology-Oncology, Department of Internal Medicine, Medical Research Institute, Pusan National University Hospital, Pusan National University School of Medicine, Busan 49241, Republic of Korea; hojinja@hanmail.net; 6 Department of Medicine, Sungkyunkwan University School of Medicine, Samsung Medical Center, Seoul 06351, Republic of Korea; kihyunk@skku.edu (K.K.); sangeun.yoon@samsung.com (S.E.Y.); 7 Department of Oncology, Asan Medical Center, University of Ulsan College of Medicine, Seoul 05505, Republic of Korea; dhyoon@amc.seoul.kr (D.H.Y.); hwcho@amc.seoul.kr (H.C.); 8 Division of Hematology/Oncology, Department of Internal Medicine, Korea Cancer Center Hospital, Korea Institute of Radiological and Medical Sciences, Seoul 01812, Republic of Korea; hyejin@kirams.re.kr; 9 Department of Internal Medicine, Seoul National University Hospital, Seoul National University College of Medicine, Seoul 03080, Republic of Korea; jaminbyun@snu.ac.kr; 10 Department of Hematology-Oncology, Ulsan University Hospital, University of Ulsan College of Medicine, Ulsan 44033, Republic of Korea; jcjo@uuh.ulsan.kr; 11 Department of Hematology-Oncology, Wonkwang University Hospital, Iksan 54538, Republic of Korea; pepupa80@wku.ac.kr; 12 Department of Internal Medicine, Hemato-Oncology, Inje University Busan Paik Hospital, Busan 47392, Republic of Korea; 103198@paik.ac.kr; 13 Department of Hematology-Oncology, Chonnam National University Hwasun Hospital, Chonnam National University Medical School, Hwasun 58128, Republic of Korea; drjejung@chonnam.ac.kr (J.-J.L.); shglory@jnu.ac.kr (S.-H.J.); 14 Division of Hematology/Oncology, Department of Internal Medicine, Chungnam National University College of Medicine, Daejeon 35015, Republic of Korea; iyoo23@cnu.ac.kr; 15 Division of Hematology-Oncology, Department of Medicine, Chung-Ang University, Seoul 06973, Republic of Korea; xuno@daum.net; 16 Department of Statistics, Dongguk University, Seoul 04620, Republic of Korea; juhyunp@dongguk.edu; 17 Department of Hematology, Seoul St. Mary’s Hospital, College of Medicine, The Catholic University of Korea, Seoul 06591, Republic of Korea

**Keywords:** multiple myeloma, relapsed, refractory, autologous stem cell transplantation, salvage therapy, second transplantation, novel agents

## Abstract

Background: Second salvage autologous stem cell transplantation (SAT) is a therapeutic option for patients with multiple myeloma (MM) who relapse after a first autologous stem cell transplantation (ASCT) in the era of novel agents. However, the clinical context in which SAT provides benefit relative to contemporary salvage regimens remains unclear. Methods: We retrospectively analyzed 51 patients who underwent SAT after novel agent-based induction and first ASCT, and salvage re-induction, and compared outcomes with 113 patients treated with salvage carfilzomib–lenalidomide–dexamethasone (KRd) without SAT. Results: Median interval from first ASCT to relapse was 27 months. In the SAT cohort, median progression-free survival (PFS) and overall survival (OS) from initiation of salvage therapy were 30 and 99 months, respectively. A time to relapse ≥18 months after first ASCT and receipt of SAT as second-line of therapy were associated with significantly longer PFS and OS. In multivariate analysis, administration of SAT at later lines was independently associated with inferior outcomes, while a time to relapse ≥18 months after first ASCT was associated with significantly longer OS. Compared with the KRd-only cohort, SAT was associated with longer OS, whereas PFS was numerically longer without statistical significance. Among patients who had received both a proteasome inhibitor and an immunomodulatory drug as salvage induction, SAT was associated with longer PFS and OS. Conclusions: SAT may provide clinical benefit in selected patients with MM, particularly those with a durable response to first ASCT and those undergoing SAT at an earlier line of relapse in the novel agent era.

## Simple Summary

Multiple myeloma is a blood cancer in which most patients eventually relapse after initial treatment, including a first autologous stem cell transplantation. Although many new drugs are now available, it remains unclear whether a second salvage autologous stem cell transplantation (SAT) still provides benefit in current clinical practice. In this study, we examined outcomes of patients who received SAT after relapse and compared them with patients treated with a commonly used drug-based salvage regimen without transplantation. We found that patients who had a long-lasting response after their first transplantation and who received an SAT early after relapse tended to have better survival outcomes. These results suggest that SAT may still be a useful treatment option for carefully selected patients, rather than a routine approach for all relapsed patients. This study helps to clarify when SAT may remain relevant in the era of effective modern therapies.

## 1. Introduction

Multiple myeloma (MM) constitutes roughly 1–2% of all cancer diagnosed and represents a significant proportion of hematologic neoplasms, at approximately 17% [1]. Although the treatment landscape has been revolutionized by the advent of novel agents over the last twenty years, MM is still considered an incurable condition. As a result, autologous stem cell transplantation (ASCT) remains a cornerstone of therapy for candidates eligible for the procedure.

ASCT was demonstrated to improve survival outcomes in patients with MM in the early 1990s. Over the past three decades, the clinical utility of ASCT has been consistently upheld, even with the introduction of novel agents. The IFM2009 trial showed that patients receiving upfront ASCT after RVD (lenalidomide, bortezomib, and dexamethasone) induction had higher complete response (CR) rates (59% vs. 48%, *p* = 0.03) and significantly longer progression-free survival (PFS) (50 months vs. 36 months, *p* < 0.001) than those who did not, with minimal transplant-related mortality [2]. Continued improvements in transplant-related mortality have established ASCT as a relatively safe treatment option, even for elderly patients [3].

However, despite these treatments, many patients still experience relapse after ASCT. Consequently, several studies have evaluated the outcomes of a second salvage ASCT (SAT) in this setting. In the BSBMT/UKMF Myeloma X trial, 297 patients who relapsed after first-line ASCT were randomized to either receive SAT with high-dose melphalan following VAD (bortezomib, doxorubicin, and dexamethasone) re-induction therapy or to receive weekly oral cyclophosphamide without SAT [4]. The results demonstrated a significant difference in PFS, with 19 months in the SAT group versus 11 months in the non-SAT group. Median overall survival (OS) also showed a statistically significant difference, with 67 months versus 52 months, confirming the clinical utility of SAT. Our group, the Korean Multiple Myeloma Working Party (KMMWP), also conducted a comparative analysis in 2013 to evaluate the real-world outcomes of SAT [5]. In this study, 48 patients who underwent SAT were matched with 144 patients who received salvage chemotherapy alone. The results showed that the SAT group had significantly improved PFS (18.0 months vs. 9.1 months, *p* = 0.017) and OS (55.5 months vs. 25.4 months, *p* = 0.035). Furthermore, this study identified factors associated with the benefit of SAT, concluding that time to progression after the first ASCT (<18 months), International Staging System (ISS)-III, and the performance of SAT were significant predictors of improved outcomes. However, in that study, 81% of patients had received VAD as induction therapy, while only 4% had undergone induction therapy that included both bortezomib and lenalidomide. Consequently, only 35% of patients achieved CR following first ASCT, which likely differs from outcomes seen in patients receiving novel agent-based induction therapies. Accordingly, prior studies do not sufficiently inform the role of SAT in patients treated with contemporary induction and salvage regimens.

Therefore, the present study aimed to investigate the clinical context in which SAT may be associated with favorable outcomes in patients who relapse after ASCT following novel agent-based induction therapy. In addition, given the widespread use of carfilzomib-based regimens as second-line treatment, outcomes after SAT were examined in relation to patients treated with carfilzomib, lenalidomide, and dexamethasone (KRd) as a contemporary reference cohort. Through this approach, this study sought to clarify the clinical relevance of SAT within current treatment paradigms.

## 2. Materials and Methods

### 2.1. Patients

This multicenter, retrospective study collected data from 164 patients across 16 institutions affiliated with the KMMWP. Among them, 51 patients underwent SAT, while 113 patients received KRd treatment without SAT following relapse. The inclusion criteria were patients diagnosed with MM through bone marrow examination between 2001 and 2020, with evaluable response and follow-up data. For the SAT group, eligible patients were those who received novel agent-based induction therapy (including bortezomib, thalidomide, and/or lenalidomide), followed by ASCT, but subsequently experienced relapse or refractory disease, underwent salvage re-induction therapy, and proceeded with SAT. For the KRd without SAT group, patients similarly received novel agent-based induction therapy and ASCT but, after relapse or refractory disease confirmed before the age of 70 (the age threshold for SAT eligibility in Korea), were treated with KRd for salvage therapy without undergoing SAT. Patients diagnosed with amyloidosis or monoclonal gammopathy of undetermined significance or those who underwent a second ASCT as tandem transplantation were excluded from the study.

### 2.2. Objectives and Definitions

The main objective was to assess PFS; secondary objectives included OS. PFS and OS were determined from the date of salvage therapy initiation to the date of disease progression or death, and to the date of death from any cause, respectively. For survival analysis, data were censored at the time of the last follow-up for patients who did not experience an event. The severity of toxicities during SAT was evaluated based on the Common Terminology Criteria for Adverse Events (CTCAE) version 5.0.

### 2.3. Statistical Analysis

To analyze categorical variables, the chi-square test or Fisher’s exact test was applied as appropriate. Survival analysis was conducted using the Kaplan–Meier method with log-rank testing to compare groups. We estimated hazard ratios (HRs) and 95% confidence intervals (CIs) using univariate and multivariate Cox proportional hazards models. Continuous variables were compared between the SAT and KRd cohorts using the *t*-test or the Mann–Whitney U test based on distribution patterns. R software (version 4.4.2; R Foundation for Statistical Computing, Vienna, Austria) was used for all analyses, and a *p*-value of <0.05 (two-sided) was considered significant.

## 3. Results

### 3.1. Patient Characteristics

We first analyzed the characteristics of all 51 patients who underwent SAT (Table 1). The median age at diagnosis was 54 years (range 39–64), and two-thirds of the patients were male. Approximately 30% of the patients were classified as ISS-III at diagnosis, and 28.1% (9/32) were identified as cytogenetic high risk, defined by the presence of t(4;14), t(14;16), or 17p deletion. All patients received an initial treatment regimen that included at least one immunomodulatory drug (IMiD) or bortezomib, with around 40% of patients treated with a combination of bortezomib, thalidomide, and dexamethasone (VTD). Before the first ASCT, 32% of patients achieved a CR, and 84% received high-dose melphalan conditioning for the initial transplantation. Subsequently, approximately 46% of the patients underwent maintenance therapy, with the majority (77.3%) undergoing thalidomide-based regimens.

The median time to relapse after the first ASCT was 26.9 months, with 16 patients (31.4%) experiencing relapse within 18 months. Regarding salvage re-induction chemotherapy, 21 patients (41.2%) received both an IMiD and proteasome inhibitor (PI). Before undergoing SAT, 34% of patients had achieved a CR. The median line of treatment (LOT) at which SAT was performed was 2 (range 2–8), and 18 patients (35.3%) underwent SAT at the third line or later. The median interval between the initiation of the salvage line of therapy and SAT was 7.2 months (range, 1.4–36.5 months). Following SAT, 21 patients (41.2%) received maintenance therapy, with thalidomide being the most commonly used agent (76.2%).

### 3.2. Survival Outcomes and Risk Factors of SAT in All Patients

**Figure 1:**
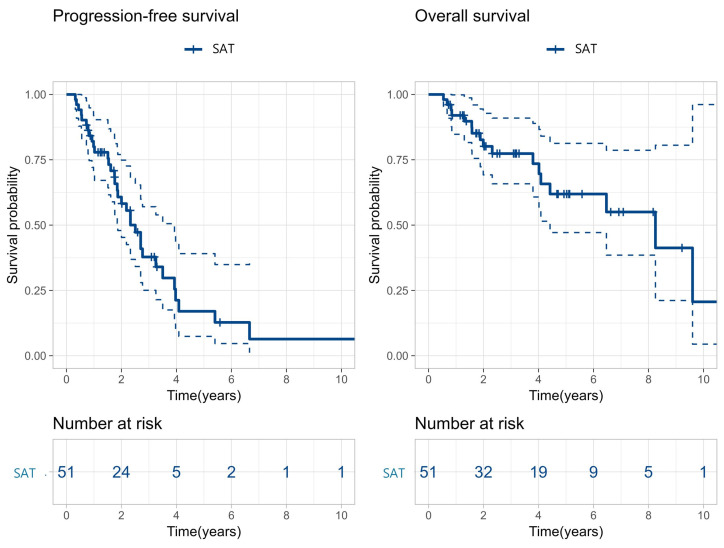
Kaplan–Meier curves for progression-free survival and overall survival in patients who received second salvage autologous stem cell transplantation. The solid lines represent the Kaplan–Meier survival estimates, and the dashed lines represent the corresponding 95% confidence intervals. SAT indicates second salvage autologous stem cell transplantation.

The median follow-up duration after SAT was 56 months, during which the median PFS and OS were 30 months and 99 months, respectively (Figure 1).

**Figure 2:**
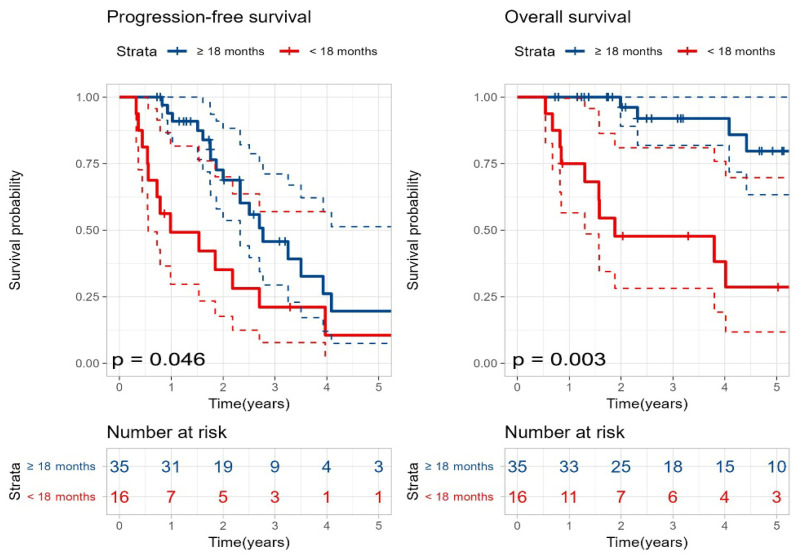
Kaplan–Meier curves for progression-free survival and overall survival after second salvage autologous stem cell transplantation according to the time to relapse after the first transplantation. Blue curves indicate relapse ≥ 18 months and red curves indicate relapse < 18 months. Dashed lines represent 95% confidence intervals. *p*-values were calculated using the log-rank test.

Survival outcomes after SAT were comparable regardless of whether patients attained a CR following their first ASCT (Figure S1). In contrast, stratifying patients by the remission duration revealed a significant survival advantage for late relapses; those who relapsed more than 18 months after the initial transplant demonstrated superior PFS (33 months vs. 12 months, *p* = 0.046) and OS (99 months vs. 23 months, *p* = 0.003) relative to the group relapsing within 18 months (Figure 2).

**Figure 3:**
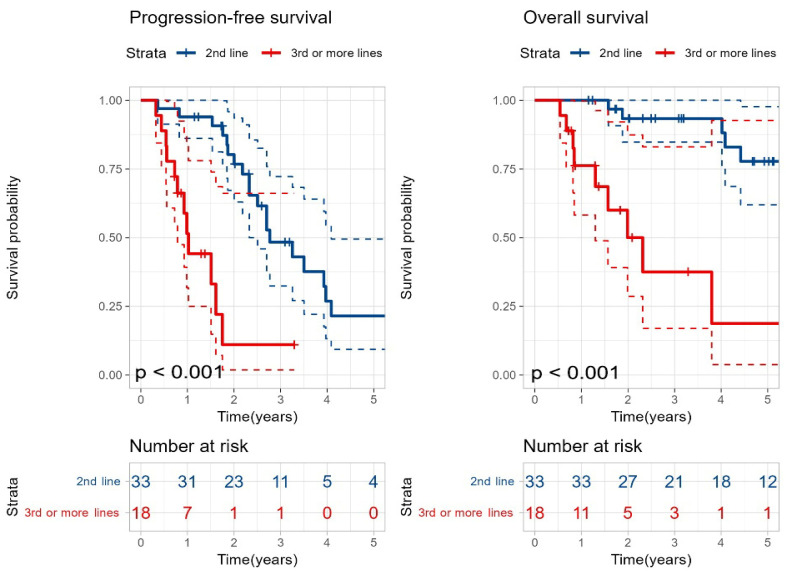
Kaplan–Meier curves for progression-free survival and overall survival based on the line of treatment at which second salvage autologous stem cell transplantation was performed. Blue curves indicate second-line SAT and red curves indicate third-line or later SAT. Dashed lines represent 95% confidence intervals. *p*-values were calculated using the log-rank test.

Additionally, comparison of outcomes based on the LOT at which SAT was performed showed that patients who underwent SAT at the 2nd line had significantly better PFS (33 months vs. 12 months, *p* < 0.001) and OS (115 months vs. 26 months, *p* < 0.001) compared to those who received SAT at the 3rd line or later (Figure 3).

**Figure 4:**
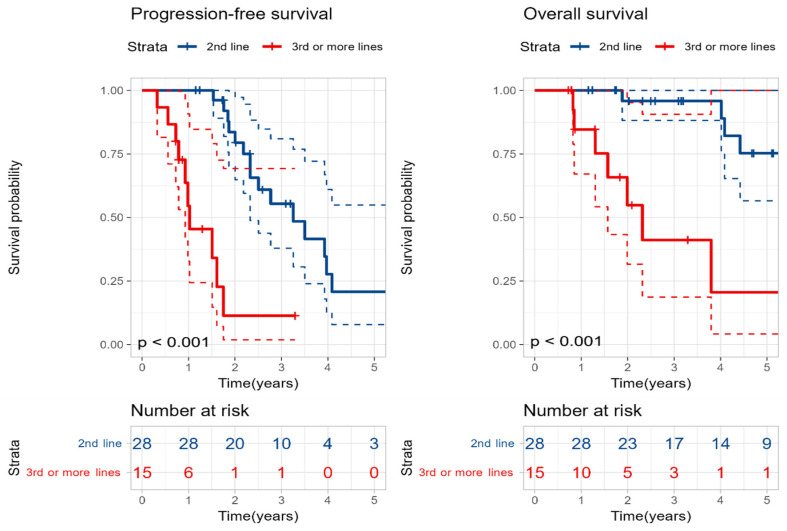
Kaplan–Meier curves for progression-free survival and overall survival based on the line of treatment at which second salvage autologous stem cell transplantation was performed in patients who achieved at least partial response just before the transplantation. Color coding and statistical methods are as described in Figure 3.

We considered the possibility that the difference in outcomes according to the LOT might be influenced by the response to salvage re-induction chemotherapy. Therefore, we compared patients who achieved CR after first salvage re-induction therapy with those who did not. However, no statistically significant difference was observed between the two groups (Figure S2). Nevertheless, when analyzing only the 43 patients who achieved partial response (PR) or better after salvage re-induction therapy, those who underwent SAT at the 2nd line demonstrated significantly better PFS (39 months vs. 12 months, *p* < 0.001) and OS (99 months vs. 28 months, *p* < 0.001) compared to those who received SAT at the 3rd line or later (Figure 4).

We further analyzed which salvage re-induction regimens were most effective in achieving a response of PR or better. The results showed that the 19 patients who received a combination of IMiD and PI had significantly higher CR rates (68.4% vs. 35.0%) and overall response rates (100% vs. 88.9%) compared to the remaining 27 patients (Figure S3 and Table S1). The administration of maintenance therapy (n = 21) did not result in statistically superior outcomes compared to observation (n = 30); median PFS (28 months vs. 30 months, *p* = 0.225) and OS (115 months vs. 99 months, *p* = 0.257) were comparable between the two groups (Figure S4). Focusing on the 26 patients who underwent SAT as a second-line therapy after a remission duration of >18 months, the median PFS and OS were 39 months and 99 months, respectively. In this subgroup, the median time from salvage therapy initiation to SAT was 9.5 months. Outcomes were particularly favorable in the 12 patients within this category who received re-induction with both an IMiD and a PI; the achieved a median PFS of 47 months and a median OS of 115 months, with a median interval of 14.6 months to transplantation.

We applied a Cox proportional hazard model to identify factors influencing survival outcomes after SAT (Table 2). The analysis revealed that performing SAT at the 3rd line or later (HR 5.677, 95% CI 2.452–13.15, *p* < 0.001) was significantly associated with shorter PFS. When assessing the impact of the same factors on OS, time to relapse within 18 months after the first ASCT (HR 4.046, 95% CI 1.511–10.83, *p* = 0.005) and performing SAT at the 3rd line or later (HR 8.121, 95% CI 2.701–24.40, *p* < 0.001) were found to be significantly associated with worse outcomes. In the multivariate analysis, performing SAT at the 3rd line or later was significantly associated with worse PFS (HR 4.518, 95% CI 1.860–10.97, *p* = 0.001) and OS (HR 7.261, 95% CI 2.206–23.89, *p* = 0.001). Time to relapse within 18 months following the first ASCT was a statistically significant adverse factor for OS (HR 3.355, 95% CI 1.207–9.325, *p* = 0.020).

### 3.3. Safety of the First ASCT and SAT

Table 3 compares the non-hematologic toxicities observed during the first ASCT and SAT. Nausea was the most common adverse event of any grade in both the first ASCT (78.7%) and SAT (77.1%). Diarrhea, vomiting, and febrile neutropenia were also relatively common adverse events. For grade 3 or higher toxicities, febrile neutropenia was the most frequent event in both the first ASCT (16.0%) and SAT (21.6%), followed by nausea. Overall, the frequency of non-hematologic toxicities between the first ASCT and SAT did not show significant differences.

During the follow-up period after SAT, a total of 17 deaths were noticed. Of these, 8 were attributed to disease progression, followed by infection (n = 4), multiorgan failure (n = 1), and 4 cases of unknown causes.

### 3.4. Comparative Analyses of SAT and KRd Without SAT

Furthermore, we examined outcomes of SAT in relation to those of KRd-treated patients, the most commonly used 2nd-line treatment in Korea, without undergoing SAT. To minimize potential bias, the SAT group was limited to 33 patients who received SAT as a 2nd-line treatment, and the KRd group (n = 113) included only patients under the age of 70, which is the approved age for ASCT eligibility in Korea.

A comparison of characteristics between the two groups showed that the SAT group was slightly younger at diagnosis (55 vs. 57 years) and had an earlier diagnosis year (2013 vs. 2016) compared to the KRd group (Table S2). The SAT group also had a lower proportion of patients who received both IMiD and PI during induction compared to the KRd group (36.4% vs. 75.2%). However, the time from diagnosis to relapse was significantly longer in the SAT group (44 months vs. 30 months). There were no significant differences between the two groups in other key prognostic factors, including ECOG performance status, ISS stage, cytogenetic risk profile, and renal function.

**Figure 5:**
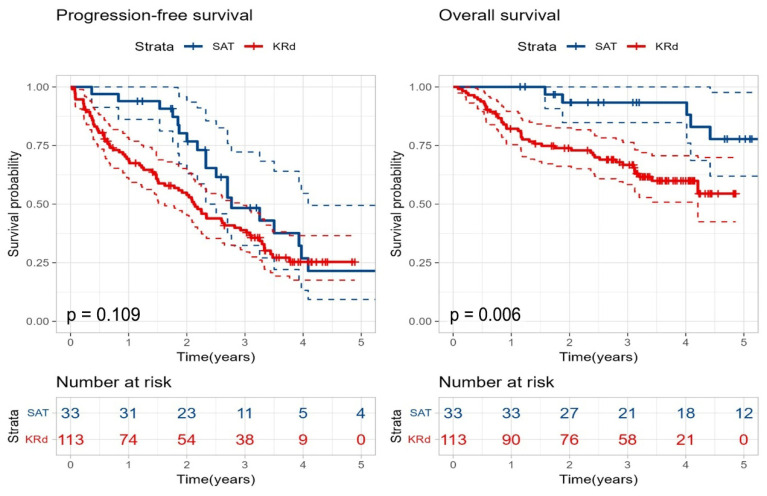
Kaplan–Meier curves for progression-free survival and overall survival in the second salvage autologous stem cell transplantation group compared with the KRd group. Blue curves indicate SAT and red curves indicate KRd. Dashed lines represent 95% confidence intervals. *p*-values were calculated using the log-rank test.

The median follow-up duration was 59 months for the SAT group and 43 months for the KRd group. A comparison of survival rates between the two groups showed that the SAT group had a trend toward better PFS, although not statistically significant (33 months vs. 25 months, *p* = 0.109), and significantly better 5-year OS (77.8% vs. 54.5%, *p* = 0.006) (Figure 5).

**Figure 6:**
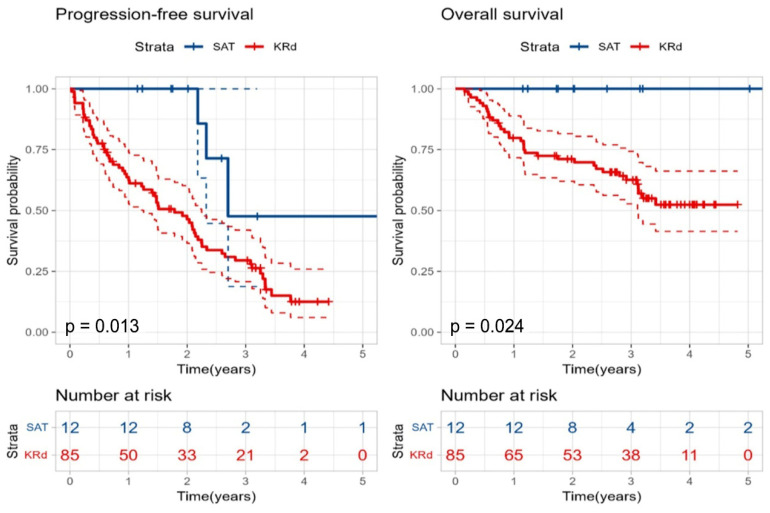
Kaplan–Meier curves for progression-free survival and overall survival in the second salvage autologous stem cell transplantation group to the KRd group in patients receiving both immunomodulatory drug and proteasome inhibitor during induction therapy. Color coding and statistical methods are as described in Figure 5.

Considering the differences in induction regimens between the two groups, we performed a separate analysis of the 97 patients who received both IMiD and PI during induction (12 in the SAT group and 85 in the KRd group). The SAT group demonstrated significantly better PFS (32 months vs. 21 months, *p* = 0.013) and 5-year OS (100% vs. 52.4%, *p* = 0.024) compared to the KRd group (Figure 6). To account for potential bias, given that SAT was performed at a median of 7.2 months after salvage therapy initiation, we conducted a landmark analysis with the landmark set at 7 months. In the landmark analysis, the SAT group maintained a numerical advantage in OS compared to the KRd group, although the difference was not statistically significant (*p* = 0.057), likely due to the reduced sample size. Nevertheless, the survival curves showed a consistent trend favoring SAT (Figure S5).

We also recognized that time to relapse is a critical variable in interpreting results. Therefore, to strictly control for the potential confound effect of time to relapse, we conducted a multivariate Cox analysis, including time to relapse and SAT administration (vs. KRd without SAT) as covariates. Across the entire cohort, the time to relapse emerged as a significant predictor for both PFS and OS. In this overall analysis, SAT administration conferred a statistically significant benefit only for OS compared to the KRd group (HR 0.346, 95% CI 0.133–0.897, *p* = 0.029) (Table S3). Conversely, when limiting the analysis to the subgroup of patients treated with both an IMiD and a PI during induction, SAT was associated with a significant improvement in PFS over KRd (HR 0.302, 95% CI 0.094–0.968, *p* = 0.044) (Table S4).

## 4. Discussion

This multicenter retrospective study examined the clinical context in which SAT may remain relevant for patients with relapsed and/or refractory MM in the contemporary treatment era. Among patients who initially received novel agent-based induction therapy followed by first ASCT, the median PFS from initiation of salvage therapy including SAT was 30 months with a median OS of 99 months. Importantly, favorable outcomes associated with SAT were not observed uniformly, but were largely confined to patients who relapsed ≥ 18 months after first ASCT and those who underwent SAT at an earlier line of relapse, indicating that disease sensitivity and treatment timing are critical determinants of benefit.

This pattern provides important understanding for interpreting randomized data from the GMMG ReLApsE trial, in which SAT following lenalidomide–dexamethasone did not result in a significant PFS advantage compared with continuous lenalidomide–dexamethasone. In that trial, a substantial proportion of patients assigned to SAT were unable to proceed to transplantation because of disease progression, and the SAT arm included a higher burden of adverse disease features, including prior tandem transplantation and high-risk cytogenetics [6,7]. Although the PFS achieved with SAT was comparable to that reported in the Myeloma X trial of 19 months, the markedly improved outcomes in the control arm of GMMG ReLApsE indicate increasing efficacy of non-transplant salvage regimens. Consistent with this interpretation, real-world studies have reported median PFS of approximately 1–2 years after SAT, with improved outcomes observed in patients with prolonged remission after first ASCT and in those treated at first relapse [8,9]. These observations are concordant with our findings, in which a longer time to relapse after first ASCT was independently associated with improved outcomes following SAT. In addition, a single-center analysis by Lemieux et al. demonstrated significantly inferior survival when SAT was deferred to later lines of therapy, supporting cumulative treatment resistance as a key adverse biological determinant [10]. Our data extend these observations by demonstrating that both time to relapse after first ASCT and the line of therapy at which SAT is performed influence outcome. Patients who satisfied both conditions showed substantially more favorable survival compared with the overall cohort, whereas the absence of either factor was associated with limited benefit from SAT.

The role of post-transplant maintenance after SAT represents another important consideration. In the present cohort, maintenance therapy after SAT did not result in a statistically significant improvement in survival, which likely reflects limited patient numbers, heterogeneous practice patterns, and predominant use of thalidomide-based regimens. Therefore, our finding should be interpreted as inconclusive rather than definite evidence of inefficacy. In contrast, recent prospective studies have demonstrated improved PFS with contemporary maintenance strategies following SAT. The Myeloma XII (ACCoRD) trial reported a significant PFS benefit with ixazomib-based consolidation and maintenance, particularly in patients without prior lenalidomide maintenance [11]. Similarly, the CARFI trial demonstrated improved PFS and quality-adjusted PFS with carfilzomib-based maintenance after SAT [12]. These data suggest that the absence of systematic modern maintenance in our cohort may underestimate the durability of disease control achievable with current post-transplant approaches, while reinforcing that patient selection remains the dominant determinant of outcome. Furthermore, the therapeutic landscape has recently evolved with the introduction of T-cell redirection therapies, such as CAR-T cells and bispecific antibodies [13,14]. Although our historical cohort precludes a direct comparison with these novel agents, this study provides a valuable benchmark for SAT outcomes in the pre-T-cell therapy era, helping to define the role of high-dose chemotherapy in the current treatment paradigm.

MM is inherently incurable, making effective salvage treatments essential for improving disease control and prolonging survival. In the present study, KRd was used as a reference regimen reflecting widely adopted second-line treatment during the study period. The median PFS of 25 months observed in our KRd cohort was consistent with the ASPIRE trial and with recent real-world data from Korean patients with RRMM, supporting its validity as a benchmark for drug-based salvage therapy [15,16]. When restricted to patients receiving second-line therapy, SAT was associated with a significantly higher 5-year OS, whereas PFS was numerically longer without reaching statistical significance. After adjustment for time to relapse, and in the subgroup of patients who had received both an IMiD and a PI during induction, SAT was associated with improved PFS. Nonetheless, with the introduction of multiple new agents and modalities—including CD38 monoclonal antibodies, B-cell maturation antigen (BCMA)–targeting chimeric antigen receptor T-cell therapy, bispecific antibodies directed against BCMA, GPRC5D, and FcRH5, as well as exportin-1 inhibitors—the optimal salvage re-induction strategy has become increasingly dynamic [17,18,19,20,21,22]. In parallel, highly active regimens are moving into earlier lines of relapse: daratumumab–carfilzomib–dexamethasone achieved a median PFS of 28 months in the CANDOR study, belantamab mafodotin-based therapy achieved a median PFS of 37 months in DREAMM-7, and teclistamab–daratumumab in the MajesTEC-3 trial demonstrated marked PFS prolongation compared with established daratumumab-based regimens [23,24,25]. These advances indicate that therapeutic alternatives to SAT are becoming increasingly potent, particularly in earlier relapse. Although the limited number of patients undergoing SAT precludes definitive comparative conclusions, the observed survival patterns support a restricted, context-dependent role for SAT alongside increasingly effective pharmacologic and immune-based salvage therapies, particularly in real-world settings where access to advanced immunotherapies is constrained by cost, regulatory approval, or infrastructure.

Our analysis revealed an intriguing finding. The depth of response to salvage re-induction therapy did not statistically influence survival outcomes after SAT. This suggests that the therapeutic benefit of SAT is driven primarily by susceptibility to high-dose conventional cytotoxic chemotherapy rather than the depth of response achieved by novel agents alone. While induction therapy with PIs or IMiDs is essential to reduce tumor burden and demonstrate that the disease is not refractory, the conditioning regimen operates through a distinct alkylating mechanism. It appears that high-dose cytotoxic chemotherapy acts as a powerful consolidation capable of eradicating residual clones. Therefore, in the context of SAT, chemosensitivity should be interpreted as the retained responsiveness to dose-intensive cytotoxic agents, which remains a crucial salvage mechanism even in the era of novel agents.

Several limitations warrant consideration. First, the retrospective design introduces heterogeneity in the induction, post-transplant management, and salvage approaches, as well as the potential for residual confounding. Although this analysis focused on patients who received novel agent-based induction before first ASCT, more contemporary regimens, including RVd and daratumumab-based quadruplets, were underrepresented [2,26,27,28,29]. Another major limitation is the small sample size in the subgroup analyses, particularly when stratifying patients by specific treatment sequences. Consequently, the favorable outcomes observed in these small subgroups should be interpreted with caution and regarded as exploratory rather than definitive. Second, given the timing of SAT after salvage initiation, time-dependent bias and residual confounding by indication cannot be fully excluded. Third, cytogenetic and minimal residual disease (MRD) data were incomplete, limiting biologic risk stratification beyond conventional clinical variables. Specifically, a substantial proportion of FISH data was missing, which limited our ability to accurately analyze the impact of high-risk cytogenetic biology on SAT outcomes. These factors constrain generalizability but also reflect real-world practice during the study period and represent a shared limitation of many real-world studies, although MRD remains a validated surrogate prognostic marker [30]. Despite these limitations, it is notable that selected patients achieved prolonged survival following SAT even in the absence of systematic post-transplant maintenance. This finding suggests that the observed outcomes are unlikely to be explained solely by treatment intensification and instead reflect patient selection and underlying disease biology. Consistent with evidence from recent trials demonstrating the continued clinical value of ASCT in the era of intensified induction especially in the high-risk group of patients, including quadruplet-based regimens, these data support a context-dependent role for SAT. Finally, the evolving landscape of myeloma therapy necessitates a re-evaluation of patient selection for SAT. Recent randomized trials suggest that the relative benefit of SAT is narrowing as novel agent-based therapies become more potent. Therefore, we propose that the selection criteria for SAT in the modern era must be more stringent than in the past. While a remission duration of 18–24 months was historically sufficient to consider a second transplant, the widespread use of highly effective quadruplet regimens and continuous therapy suggests that the threshold should likely be raised. Future guidelines may need to define ‘SAT-eligible’ candidates as those achieving a significantly prolonged first remission (e.g., time to relapse > 36–48 months) to maximize the cost-benefit ratio of this intensive procedure. In this context, our study serves as a historical reference, highlighting that even in the absence of modern immunotherapies, long-term remission was the strongest predictor of SAT success.

In conclusion, this study suggests that SAT may retain a role in the novel agent era when applied selectively. The association between SAT and favorable outcomes appears to depend on disease sensitivity and treatment timing rather than on the procedure itself. In an era shaped by immune-based salvage therapies, these findings help define a narrower, context-specific role for SAT and provide a framework for patient selection in future studies incorporating modern maintenance and immunotherapeutic approaches.

## Figures and Tables

**Table 1:** Baseline characteristics of patients who received second salvage autologous stem cell transplantation.

Characteristics	Number (%)
Sex	
male	34 (66.7%)
female	17 (33.3%)
Age at diagnosis (y.o.) *	54 (39–64)
Year of diagnosis *	2014 (2005–2019)
Immunochemical type	
secretory	49 (96.1%)
non-secretory	2 (3.9%)
Heavy chain	
IgG	28/50 (56.0%)
IgA	10/50 (20.0%)
IgD	1/50 (2.0%)
Light chain only	11/50 (22.0%)
Light chain	
kappa	30/50 (60.0%)
lambda	20/50 (40.0%)
ECOG PS	
0–1	45/51 (88.2%)
>1	6/51 (11.8%)
ISS	
I	19/47 (40.4%)
II	14/47 (29.8%)
III	14/47 (29.8%)
BM plasma cell at diagnosis (%)	40.0 (0.3–100)
Karyotype at diagnosis	
normal	26/42 (61.9%)
abnormal (non-complex)	3/42 (7.1%)
abnormal (complex)	13/42 (31.0%)
FISH risk stratification	
standard risk	23/32 (71.9%)
high risk ^†^	9/32 (28.1%)
FISH results	
17p del	6/32 (18.8%)
t(4;14)	5/32 (15.6%)
t(11;14)	6/31 (19.4%)
t(14;16)	2/29 (6.9%)
1q gain	5/24 (20.8%)
1p del	0/10 (0.0%)
Extramedullary mass (present)	7/51 (13.7%)
Serum albumin	
<3.5 g/dL	23/50 (46.0%)
≥3.5 g/dL	27/50 (54.0%)
Serum creatinine	
<1.5 mg/dL	38/49 (77.6%)
≥1.5 mg/dL	11/49 (22.4%)
Beta-2-microglobulin	
<3.5 mg/L	23/47 (48.9%)
≥3.5–5.5 mg/L	10/47 (21.3%)
≥5.5 mg/L	14/47 (29.8%)
LDH	
normal	33/46 (71.7%)
elevated	13/46 (28.3%)
Induction	
IMiD-based	19/51 (37.3%)
PI-based	12/51 (23.5%)
IMiD + PI	20/51 (39.2%)
Induction regimen	
VTD	20/51 (39.2%)
TD	9/51 (17.6%)
CTD	7/51 (13.7%)
VD	6/51 (11.8%)
PAD	4/51 (7.8%)
other	5/51 (9.8%)
Pre-1st ASCT response	
CR	16/50 (32.0%)
VGPR	18/50 (36.0%)
PR	13/50 (26.0%)
SD	2/50 (4.0%)
MR	1/50 (2.0%)
Mobilization	
G-CSF only	15/51 (29.4%)
G-CSF with cyclophosphamide	23/51 (45.1%)
G-CSF with etoposide	2/51 (3.9%)
G-CSF with other agent	11/51 (21.6%)
Conditioning regimen for 1st ASCT	
HD MEL	42/50 (84.0%)
BUMEL	6/50 (12.0%)
BUTHIO	1/50 (2.0%)
Other	1/50 (2.0%)
Infused cell dose for 1st ASCT (×10^6^/kg) *	6.40 (2.5–17.3)
Response after 1st ASCT	
CR	34/48 (70.8%)
VGPR	8/48 (16.7%)
PR	3/48 (6.3%)
SD	3/48 (6.3%)
Maintenance after 1st ASCT (done)	22/48 (45.8%)
Maintenance regimen after 1st ASCT	
Thalidomide	17/22 (77.3%)
Lenalidomide	0/22 (0.0%)
Ixazomib	1/22 (4.5%)
Bortezomib	1/22 (4.5%)
other	3/22 (13.6%)
Time to relapse from 1st ASCT (months) *	26.9 (2.2–117.1)
<18 months	16/51 (31.4%)
≥18 months	35/51 (68.6%)
BM plasma cell at relapse (%) *	11.4 (1.2–77.0)
Karyotype at relapse	
normal	21/25 (84.0%)
abnormal (non-complex)	0/25 (0.0%)
abnormal (complex)	4/25 (16.0%)
FISH risk stratification at relapse	
standard risk	7/16 (43.8%)
high risk ^†^	9/16 (56.2%)
17p del	6/14 (42.9%)
t(4;14)	4/14 (28.6%)
t(11;14)	3/16 (18.8%)
t(14;16)	3/13 (23.1%)
1q gain	4/13 (30.8%)
1p del	0/5 (0.0%)
Extramedullary mass at relapse (present)	5/51 (9.8%)
Salvage chemotherapy after relapse	
IMiD-based	8/51 (15.7%)
PI-based	21/51 (41.2%)
IMiD + PI	21/51 (41.2%)
other	1/51 (2.0%)
Salvage regimen after relapse	
KRd	16/51 (31.4%)
VD	13/51 (25.5%)
CTD	5/51 (9.8%)
IRD	3/51 (5.9%)
other	14/51 (27.5%)
Response before SAT	
CR	17/50 (34.0%)
VGPR	12/50 (24.0%)
PR	14/50 (28.0%)
SD	2/50 (4.0%)
PD	5/50 (10.0%)
Year of SAT *	2019 (2006–2023)
LOT of SAT *	2 (2–8)
2	33/51 (64.7%)
>2	18/51 (35.3%)
Age at SAT (y.o.) *	59 (45–72)
≤60	31/51 (60.8%)
>60	20/51 (39.2%)
Interval between 1st ASCT and SAT (months) *	51 (7–124)
Conditioning regimen for SAT	
HD MEL	42/51 (82.4%)
BUMEL	6/51 (11.8%)
other	3/51 (5.9%)
Infused cell dose for SAT (×10^6^/kg) *	5.09 (2.55–237.5)
Response after SAT	
CR	29/50 (58.0%)
VGPR	7/50 (14.0%)
PR	9/50 (18.0%)
SD	2/50 (4.0%)
MR	2/50 (4.0%)
PD	1/50 (2.0%)
Maintenance after SAT (done)	21/51 (41.2%)
Maintenance regimen after SAT	
Thalidomide	16/21 (76.2%)
Lenalidomide	4/21 (19.0%)
Ixazomib	0/21 (0%)
Bortezomib	0/21 (0%)
other	1/21 (4.8%)

* Presented as median (range), ^†^ defined as t(4;14), t(14;16), or 17p del. ECOG PS, Eastern Cooperative Oncology Group performance status; ISS, International Staging System; BM, bone marrow; FISH, fluorescence in situ hybridization; LDH, lactate dehydrogenase; IMiD, immunomodulatory drug; PI, proteasome inhibitor; VTD, bortezomib, thalidomide, and dexamethasone; TD, thalidomide, dexamethasone; CTD, cyclophosphamide, thalidomide, and dexamethasone; VD, bortezomib, dexamethasone; PAD, bortezomib, doxorubicin, and dexamethasone; ASCT, autologous stem cell transplantation; CR, complete remission; VGPR, very good partial response; PR, partial response; SD, stable disease; MR, minimal response; G-CSF, granulocyte colony-stimulating factor; HD MEL, high-dose melphalan; BUMEL, busulfan, melphalan; BUTHIO, busulfan, thiotepa; KRd, carfilzomib, lenalidomide, and dexamethasone; IRD, ixazomib, lenalidomide, and dexamethasone; LOT, line of treatment; SAT, second salvage autologous stem cell transplantation; PD, progressive disease. ^†^ Data derived from 27 patients who underwent bone marrow biopsy at relapse.

**Table 2:** Univariate and multivariate analyses of factors influencing progression-free survival and overall survival after second salvage autologous stem cell transplantation.

	Univariate Cox Analysis						Multivariate Cox Analysis					
Variable	PFS			OS			PFS			OS		
	HR	95% CI	*p*	HR	95% CI	*p*	HR	95% CI	*p*	HR	95% CI	*p*
ISS (III vs. I or II)	1.233	0.570–2.668	0.595	2.383	0.862–6.585	0.094						
FISH risk (high vs. standard) *	0.828	0.306–2.245	0.711	0.841	0.209–3.390	0.808						
LDH (elevated vs. normal)	0.463	0.192–1.116	0.086	0.422	0.113–1.581	0.200						
Induction (PI + IMiD vs. others)	1.377	0.612–3.098	0.439	1.152	0.360–3.680	0.812						
Response after induction (CR vs. non-CR)	1.369	0.589–3.180	0.465	0.845	0.292–2.441	0.755						
Response after 1st SCT (CR vs. non-CR)	1.212	0.574–2.563	0.614	1.041	0.374–2.896	0.939						
Time to relapse after 1st SCT (>18 vs. ≥18 months)	2.021	0.998–4.090	0.051	4.046	1.511–10.83	0.005	1.272	0.587–2.757	0.542	3.355	1.207–9.325	0.020
Salvage chemotherapy (IMiD + PI vs. others)	2.257	0.973–5.238	0.058	2.085	0.585–7.433	0.257	1.727	0.721–4.137	0.220	1.100	0.288–4.203	0.889
Line of treatment for SAT (2nd line vs. 3rd or more)	5.677	2.452–13.15	<0.001	8.121	2.701–24.40	<0.001	4.518	1.860–10.97	0.001	7.261	2.206–23.89	0.001
Response after 1st-salvage chemotherapy (CR vs. non-CR)	1.540	0.699–3.392	0.284	0.901	0.317–2.561	0.846						
Response pre-SAT (CR vs. non-CR)	1.944	0.871–4.342	0.105	1.066	0.391–2.903	0.901						
Age at SAT (>60 vs. ≤60 y.o.)	0.748	0.359–1.557	0.438	0.657	0.206–2.099	0.478						
Maintenance therapy after SAT (done vs. not done)	1.578	0.747–3.331	0.232	1.829	0.634–5.278	0.264						

* defined as t(4;14), t(14;16), or 17p del. ISS, International Staging System; FISH, fluorescence in situ hybridization; LDH, lactate dehydrogenase; PI, proteasome inhibitor; IMiD, immunomodulatory drug; CR, complete response; SCT, stem cell transplantation; SAT, second salvage autologous stem cell transplantation; PFS, progression-free survival; OS, overall survival; HR, hazard ratio; CI, confidence interval.

**Table 3:** Adverse events during the first autologous stem cell transplantation and second salvage autologous stem cell transplantation.

	1st ASCT			SAT		
Event	Grade 1 & 2	Grade ≥ 3	Total	Grade 1 & 2	Grade ≥ 3	Total
Gastrointestinal adverse events						
Nausea	32/47 (68.1%)	5/47 (10.6%)	37/47 (78.7%)	31/48 (64.6%)	6/48 (12.5%)	37/48 (77.1%)
Vomiting	21/47 (44.6%)	1/47 (2.1%)	22/47 (46.8%)	19/48 (39.6%)	2/48 (4.2%)	21/48 (43.8%)
Stomatitis	6/45 (13.4%)	2/45 (4.4%)	8/45 (17.8%)	3/46 (6.5%)	2/46 (4.3%)	5/46 (10.9%)
Constipation	6/45 (13.3%)	0	6/45 (13.3%)	4/46 (8.7%)	0	4/46 (8.7%)
Diarrhea	29/46 (63.0%)	2/46 (4.3%)	31/46 (67.3%)	30/46 (65.3%)	5/46 (10.9%)	35/46 (76.2%)
Hepatobiliary adverse events						
Hyperbilirubinemia	2/50 (4.0%)	1/50 (2.0%)	3/50 (6.0%)	2/51 (3.9%)	0	2/51 (3.9%)
Elevated transaminases	16/50 (32.0%)	1/50 (2.0%)	17/50 (34.0%)	7/51 (13.7%)	1/51 (2.0%)	8/51 (15.7%)
Nephrologic adverse events						
Azotemia	7/50 (14.0%)	0	7/50 (14.0%)	1/51 (2.0%)	1/51 (2.0%)	2/51 (3.9%)
Febrile neutropenia	10/50 (20.0%)	8/50 (16.0%)	18/50 (36.0%)	14/51 (27.4%)	11/51 (21.6%)	25/51 (49.0%)

ASCT, autologous stem cell transplantation; SAT, second salvage autologous stem cell transplantation.

## Data Availability

The data presented in this study is available on request from the corresponding author. The data is not publicly available due to privacy or ethical restrictions.

## Supplementary Materials

The following supporting information can be downloaded at: https://www.mdpi.com/article/10.3390/cancers18030471/s1. Table S1: Comparison of response rates between immunomodulatory drug with proteasome inhibitor combination regimen and other regimens in salvage re-induction therapy; Table S2: Comparison of baseline characteristics be-tween the SAT group and the KRd group; Table S3: Multivariate Cox analysis of time to relapse and second salvage autologous stem cell transplantation in all patients; Table S4: Multivariate Cox analysis of time to relapse and sec-ond salvage autologous stem cell transplantation in patients who received both immunomodulatory drug and proteasome inhibitor during induction therapy; Figure S1: Kaplan–Meier curves for progression-free survival and overall survival after second salvage autologous stem cell transplantation according to the achievement of complete remission after the first transplantation; Figure S2: Kaplan–Meier curves for progression-free survival and overall survival based on response status before salvage second autologous stem cell transplantation was performed; Figure S3: Response rates to salvage re-induction therapy compared between immunomodulatory drug with proteasome inhibitor combination regimen (**a**) and other regimens (**b**); Figure S4: Kaplan-Meier curves for progression-free survival and overall survival in patients who received maintenance therapy after second salvage autologous stem cell transplantation compared to those who did not; Figure S5: Landmark analysis of progression-free survival and overall survival comparing second salvage autologous stem cell transplantation group and the KRd group.
