# Supplementary material for: Second Salvage Autologous Hematopoietic Stem Cell Transplantation in Patients with Relapsed/Refractory Multiple Myeloma in the Era of Novel Agents: Results of the KMM2301 Study
Source: Cancers (Basel). 2026 Jan 30;18(3):471. doi: 10.3390/cancers18030471 (PMC12896849; doi:10.3390/cancers18030471)
Supplement: Supplementary file 1 [file cancers-18-00471-s001.zip › cancers-4111740-supplementary.pdf]

SUPPLEMENTARY MATERIAL

Table S1. Comparison of response rates between immunomodulatory drug with proteasome inhibitor combination regimen and other regimens in salvage re-induction therapy

	**IMiD+PI***	**Others†**	**p**
**Number**	19	27	
**CR**	13 (68.4%)	7 (35.0%)	***0.014***
**VGPR**	4 (21.1%)	5 (18.5%)	
**PR**	2 (10.5%)	12 (44.4%)	
**SD**	0	2 (7.4%)	
**PD**	0	1 (3.7%)	

IMiD, immunomodulatory drug; PI, proteasome inhibitor; CR, complete remission; VGPR, very good partial response; PR, partial response; SD, stable disease; PD, progressive disease

* Immunomodulatory drug with proteasome inhibitor included KRd (n=14), IRd (n=3), and VTD (n=2)

† Other regimen included Vd (n=12), CTD (n=5), Velyx (n=2), Rd (n=2), VCD (n=2), and DRd, DVd, Kd, and bortezomib monotherapy (each n=1)

Table S2. Comparison of baseline characteristics between the SAT group and the KRd group

		SAT group	KRd without SAT group (control)	p-value
**Number**		33	113	
**Sex (male)**		21/33 (63.6%)	62/113 (54.9%)	0.428
**Age at diagnosis (y.o.)** *		55 (39-64)	57 (26-67)	***0.033***
**Year of diagnosis***		2013 (2005-2019)	2016 (2011-2019)	***<0.001***
**Immunochemical type**				0.682
	**secretory**	32/33 (97.0%)	104/111 (93.7%)	
	**non-secretory**	1/33 (3.0%)	7/111 (6.3%)	
**Heavy chain**				0.772
	**IgG**	22/33 (66.7%)	67/113 (59.3%)	
	**IgA**	5/33 (15.2%)	20/113 (17.7%)	
	**IgD**	1/33 (3.0%)	1/113 (0.9%)	
	**IgE**	0/33 (0.0%)	1/113 (0.9%)	
	**Light chain only**	5/33 (15.2%)	23/113 (20.4%)	
	**Unclassifiable**	0/33 (0.0%)	1/113 (0.9%)*	
**Light chain**				0.675
	**Kappa**	19/33 (57.6%)	59/113 (52.2%)	
	**Lambda**	14/33 (42.4%)	49/113 (43.4%)	
	**Heavy chain only**	0/33 (0.0%)	5/113 (4.4%)	
**ECOG PS at diagnosis**				1.000
	**0-1**	29/33 (87.9%)	93/108 (86.1%)	
	**>1**	4/33 (12.1%)	15/108 (13.9%)	
**ISS**				0.535

**I**	13/30 (43.3%)	35/108 (32.4%)	
**II**	10/30 (33.3%)	39/108 (36.1%)	
**III**	7/30 (23.3%)	34/108 (31.5%)	
**BM plasma cell at diagnosis (%)***	35.0 (10-100)	45.5 (0-100)	0.503
**Karyotype at diagnosis**			0.441
**normal**	18/27 (66.7%)	58/100 (58.0%)	
**abnormal (non-complex)**	2/27 (7.4%)	18/100 (18.0%)	
**abnormal (complex)**	7/27 (25.9%)	24/100 (24.0%)	
**FISH risk stratification**			1.000
**standard risk**	13/20 (65.0%)	60/89 (67.4%)	
**high risk†**	7/20 (35.0%)	29/89 (32.6%)	
**FISH results**			
**17p del**	5/21 (23.8%)	21/91 (23.1%)	1.000
**t(4;14)**	4/20 (20.0%)	15/82 (18.3%)	1.000
**t(11;14)**	5/20 (25.0%)	11/69 (15.9%)	0.342
**t(14;16)**	2/19 (10.5%)	3/78 (3.8%)	0.252
**1q gain**	4/16 (25.0%)	21/72 (29.2%)	1.000
**1p del**	0/8 (0%)	5/57 (8.8%)	1.000
**Serum creatinine (mg/dL)**	1.00 (0.56-3.89)	0.95 (0.40-8.22)	0.536
**Albumin**			0.315
**<3.5 g/dL**	17/33 (51.5%)	43/108 (39.8%)	
**≥3.5g/dL**	16/33 (48.5%)	65/108 (60.2%)	
**Serum beta 2-microglobulin**			0.568
**<3.5 mg/L**	17/30 (56.7%)	48/105 (45.7%)	
**3.5~5.5 mg/L**	6/30 (20.0%)	23/105 (21.9%)	
**≥5.5 mg/L**	7/30 (23.3%)	34/105 (32.4%)	

**LDH**			1.000
**normal**	20/29 (69.0%)	68/102 (66.7%)	
**elevated**	9/29 (31.0%)	34/102 (33.3%)	
**Induction**			***<0.001***
**IMiD-based**	15/33 (45.5%)	23/113 (20.4%)	
**PI-based**	6/33 (18.2%)	5/113 (4.4%)	
**IMiD+PI**	12/33 (36.4%)	85/113 (75.2%)	
**Induction regimen**			***<0.001***
**VTD**	12/33 (36.4%)	84/113 (74.3%)	
**TD**	6/33 (18.2%)	17/113 (15.0%)	
**CTD**	7/33 (21.2%)	4/113 (3.5%)	
**other**	8/33 (24.2%)	8/113 (7.2%)	
**Age at relapse (y.o.)** *	59 (45-72)	60 (28-69)	0.503
**≤60 y.o.**	20/33 (60.6%)	64/113 (56.6%)	0.842
**> 60 y.o.**	13/33 (39.4%)	49/113 (43.4%)	
**Duration from diagnosis to relapse (months)** *	44 (7-125)	30 (8-104)	***0.007***

*Presented as median (range), †defined as t(4;14), t(14;16), or 17p del

SAT, second salvage autologous stem cell transplantation; KRd, carfilzomib, lenalidomide, and dexamethasone; ECOG PS, Eastern Cooperative Oncology Group performance status; ISS, International Staging System; BM, bone marrow; FISH, fluorescence in situ hybridization; LDH, lactate dehydrogenase; IMiD, immunomodulatory drug; PI, proteasome inhibitor; VTD, bortezomib, thalidomide, and dexamethasone; TD, thalidomide, dexamethasone; CTD, cyclophosphamide, thalidomide, and dexamethasone

Table S3. Multivariate Cox analysis of time to relapse and second salvage autologous stem cell transplantation in all patients

	PFS			OS		
	**HR**	**95% CI**	**p**	**HR**	**95% CI**	**p**
**Time to relapse (below median, 34.1 months)**	2.738	1.771-4.232	***<0.001***	2.738	1.417-5.289	***0.003***
**SAT (vs. KRd only)**	0.754	0.454-1.254	0.277	0.346	0.133-0.897	***0.029***

PFS, progression-free survival; OS, overall survival; SAT, second salvage autologous stem cell transplantation; KRd, carfilzomib, lenalidomide, and dexamethasone

Table S4. Multivariate Cox analysis of time to relapse and second salvage autologous stem cell transplantation in patients who received both immunomodulatory drug and proteasome inhibitor during induction therapy

	PFS			OS		
	**HR**	**95% CI**	**p**	**HR**	**95% CI**	**p**
**Time to relapse (below median, 28.0 months)**	2.137	1.137-4.018	***0.018***	1.758	0.729-4.242	0.209
**SAT (vs. KRd only)**	0.302	0.094-0.968	***0.044***	NE	0.000-3.312E+174	0.952

PFS, progression-free survival; OS, overall survival; SAT, second salvage autologous stem cell transplantation; KRd, carfilzomib, lenalidomide, and dexamethasone; NE, not estimable

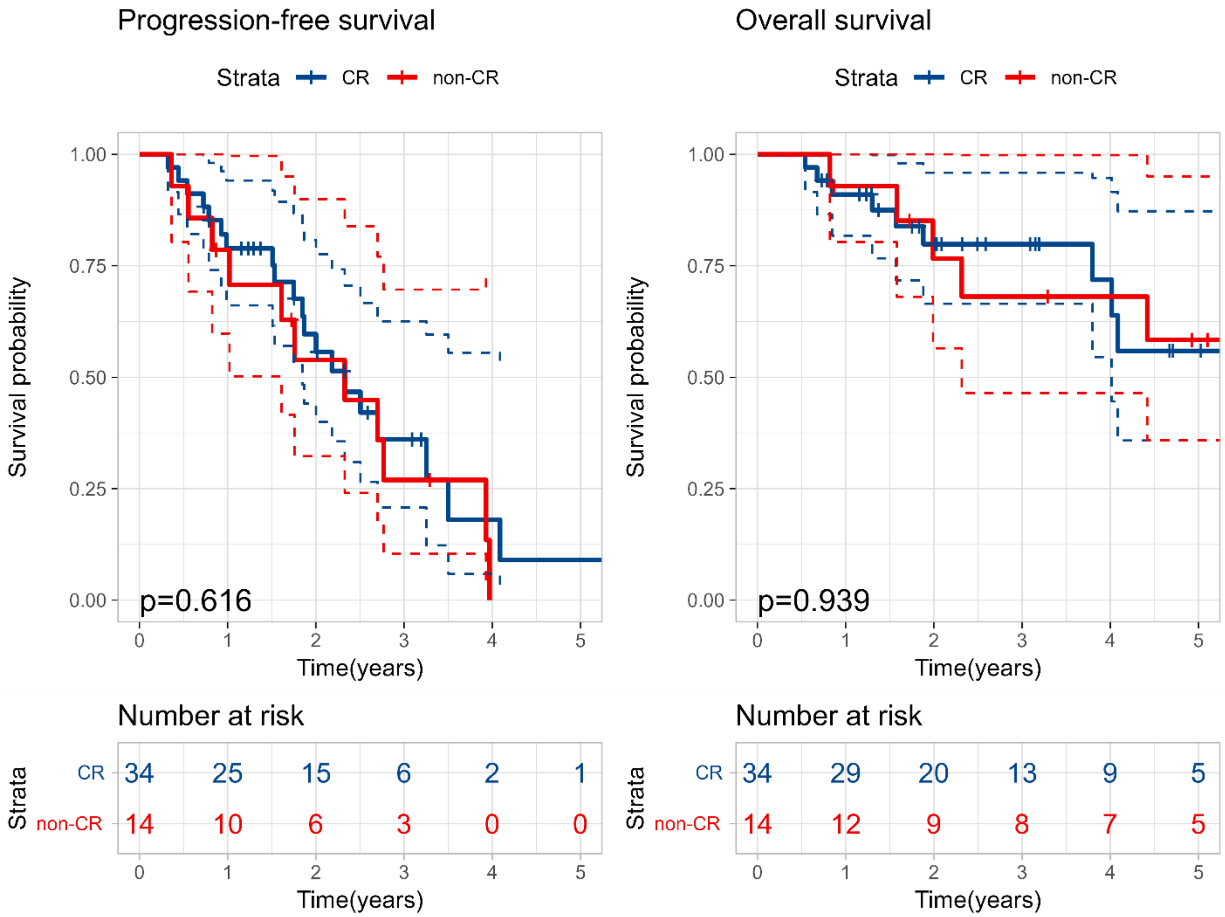

Figure S1. Kaplan–Meier curves for progression-free survival and overall survival after second salvage autologous stem cell transplantation according to the achievement of complete remission after the first transplantation.

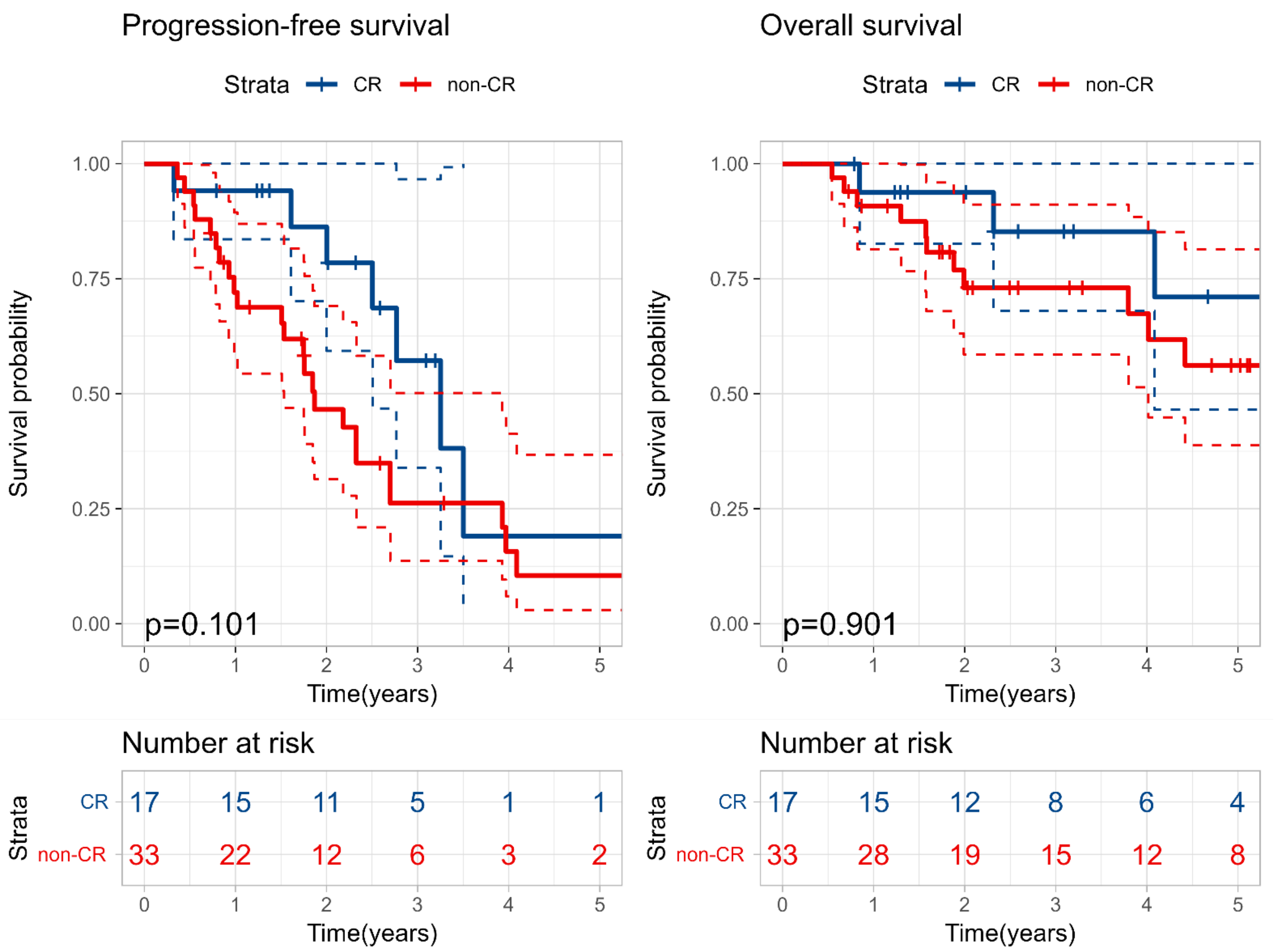

Figure S2. Kaplan–Meier curves for progression-free survival and overall survival based on response status before salvage second autologous stem cell transplantation was performed.

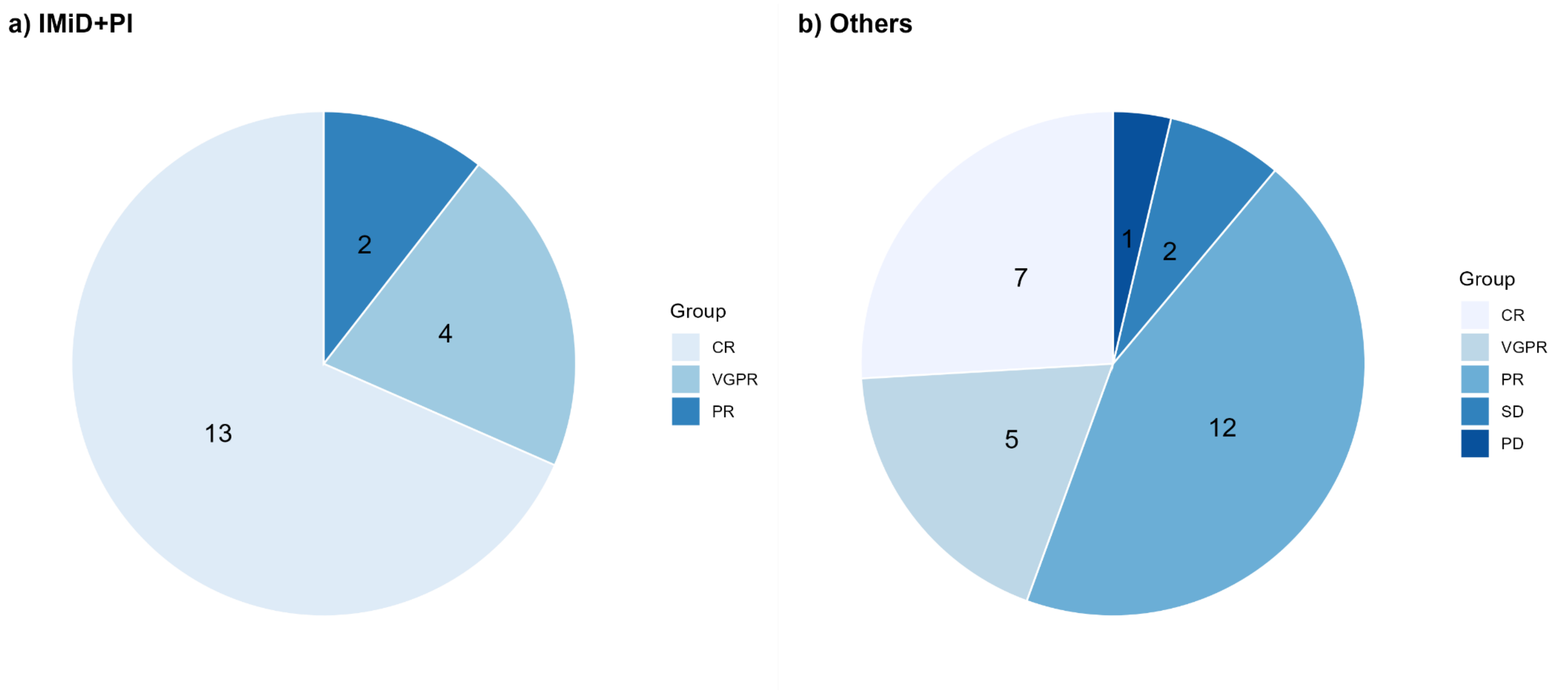

Figure S3. Response rates to salvage re-induction therapy compared between immunomodulatory drug with proteasome inhibitor combination regimen (a) and other regimens (b)

Immunomodulatory drug with proteasome inhibitor included KRd (n=14), IRd (n=3), and VTD (n=2)
Other regimen included Vd (n=12), CTD (n=5), Velyx (n=2), Rd (n=2), VCD (n=2), and DRd, DVd, Kd, and bortezomib monotherapy (each n=1)

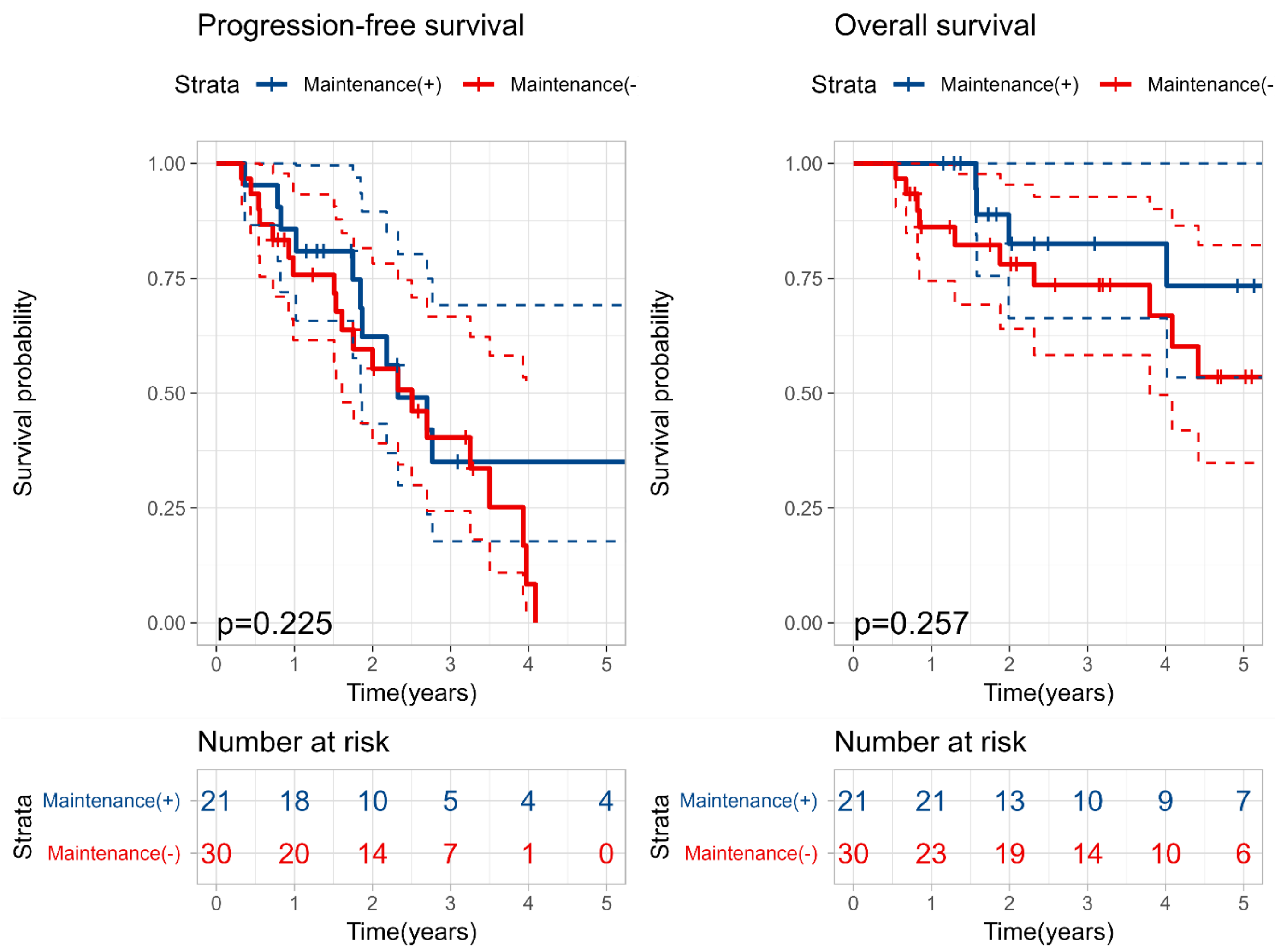

Figure S4. Kaplan-Meier curves for progression-free survival and overall survival in patients who received maintenance therapy after second salvage autologous stem cell transplantation compared to those who did not.

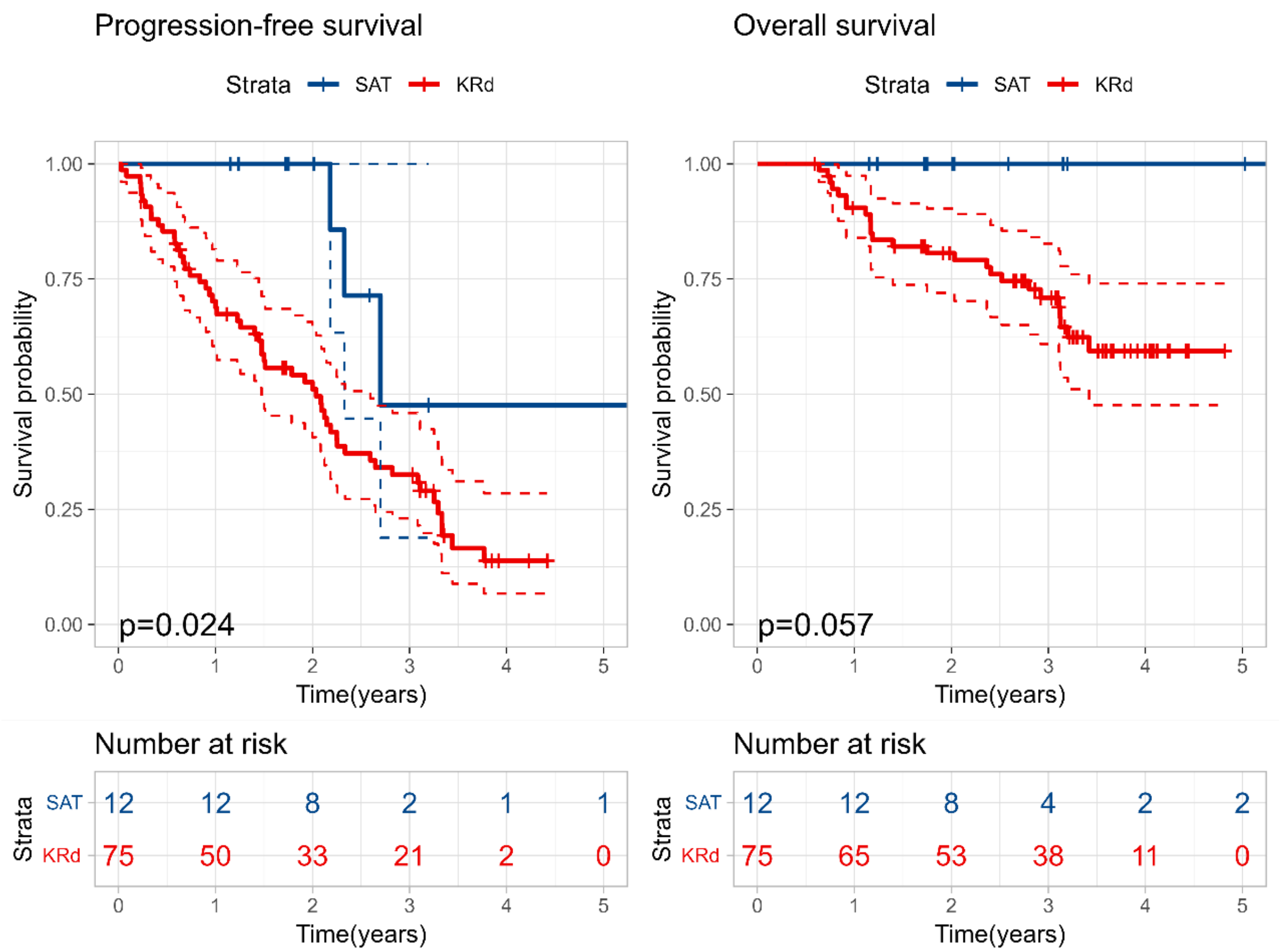

Figure S5. Landmark analysis of progression-free survival and overall survival comparing second salvage autologous stem cell transplantation group and the KRd group.
